# Counting Steps in Activities of Daily Living in People With a Chronic Disease Using Nine Commercially Available Fitness Trackers: Cross-Sectional Validity Study

**DOI:** 10.2196/mhealth.8524

**Published:** 2018-04-02

**Authors:** Darcy Ummels, Emmylou Beekman, Kyra Theunissen, Susy Braun, Anna J Beurskens

**Affiliations:** ^1^ Research Centre for Autonomy and Participation of Persons with a Chronic Illness Zuyd University of Applied Sciences Heerlen Netherlands; ^2^ ParaMedisch Centrum Zuid Sittard Netherlands; ^3^ Care and Public Health Research Institute School for Public Health and Primary Care Department of Family Medicine Maastricht University Medical Centre Maastricht Netherlands; ^4^ Research Centre for Nutrition, Lifestyle and Exercise Zuyd University of Applied Sciences Heerlen Netherlands; ^5^ Care and Public Health Research Institute School for Public Health and Primary Care Department of Health Services Research Maastricht University Medical Centre Maastricht Netherlands

**Keywords:** activity tracker, accelerometer, wearable, chronic disease, validity, physical therapy, physical activity

## Abstract

**Background:**

Measuring physical activity with commercially available activity trackers is gaining popularity. People with a chronic disease can especially benefit from knowledge about their physical activity pattern in everyday life since sufficient physical activity can contribute to wellbeing and quality of life. However, no validity data are available for this population during activities of daily living.

**Objective:**

The aim of this study was to investigate the validity of 9 commercially available activity trackers for measuring step count during activities of daily living in people with a chronic disease receiving physiotherapy.

**Methods:**

The selected activity trackers were Accupedo (Corusen LLC), Activ8 (Remedy Distribution Ltd), Digi-Walker CW-700 (Yamax), Fitbit Flex (Fitbit inc), Lumoback (Lumo Bodytech), Moves (ProtoGeo Oy), Fitbit One (Fitbit inc), UP24 (Jawbone), and Walking Style X (Omron Healthcare Europe BV). In total, 130 persons with chronic diseases performed standardized activity protocols based on activities of daily living that were recorded on video camera and analyzed for step count (gold standard). The validity of the trackers’ step count was assessed by correlation coefficients, t tests, scatterplots, and Bland-Altman plots.

**Results:**

The correlations between the number of steps counted by the activity trackers and the gold standard were low (range: –.02 to .33). For all activity trackers except for Fitbit One, a significant systematic difference with the gold standard was found for step count. Plots showed a wide range in scores for all activity trackers; Activ8 showed an average overestimation and the other 8 trackers showed underestimations.

**Conclusions:**

This study showed that the validity of 9 commercially available activity trackers is low measuring steps while individuals with chronic diseases receiving physiotherapy engage in activities of daily living.

## Introduction

The use of activity tracking to self-monitor physical activity is gaining popularity. In 2015, 1 out of 3 Dutch inhabitants was using apps, wearables, or activity trackers [1]. Physical activity is the most popular variable measured with these devices followed by nutrition, weight, and body functions (eg, blood pressure) [1]. Initially, these activity trackers were developed for athletes and the healthy population, but they could potentially also be useful in treating people with medical conditions (eg, physiotherapy treatments). The Royal Dutch Society for Physical Therapy composed physical activity intervention guidelines for the most common chronic diseases seen by a physiotherapist [2]: cardiovascular disease [3], diabetes mellitus [4], chronic obstructive pulmonary disease (COPD) [5], chronic pain [6], cancer [7], and osteoarthritis [8]. In all these guidelines, it is recommended to objectively measure the physical activity level of a patient outside of guided therapy [2]. Frequently used measurement tools by physiotherapists are questionnaires or diaries, but they have limited reliability and validity, tend to overestimate most activities while underestimating low intensity activities, and are time consuming to fill out [9,10]. For patients and physiotherapists, more objective and feasible measurement tools are useful, and activity trackers seem to be a good alternative [11].

To provide guidance in choosing an appropriate activity tracker for people with a chronic disease, we performed a literature search on the validity of activity trackers, preferably commercially available ones. The following criteria were taken into account. First, step count was considered to be the most important outcome, since it is specific to ambulation and easily interpreted by patients and physiotherapists [11]. Second, people with a chronic disease should be the target population of the study, as they often have impaired ambulatory abilities (eg, shuffling) [12], and activity trackers may measure incorrectly due to these altered walking patterns [13-15]. Third, activities of daily living should be assessed (no laboratory settings), as insight into these specific activities (eg, vacuum cleaning, walking stairs) is needed to monitor and coach participants in daily life, and activity trackers are not able to measure validly during low walk speeds (<0.8 m/s) [16], which is often the case in activities of daily living. Last, published articles were screened on standardization of the performed activities of daily living by means of an activity protocol.

Although the literature on clinometric quality of commercially available activity trackers is growing [17-19], only a few recent studies were found in which almost all criteria were met (validity of step count of commercially available activity trackers during free living conditions) [19,20]. However, the target population in those studies consisted of healthy participants.

Remoortel et al [18] recently published a literature review regarding validity and reliability of activity trackers in people with a chronic disease. It was confirmed that most commercially available activity trackers have been studied in healthy populations [17,19-21], and little is known about which types of activity trackers provide valid results in people with chronic diseases. In their review, they found that only 12 of the 134 studies on validity of activity trackers included people with a chronic disease [18]. Of the 12 identified studies, only 3 evaluated activities of daily living (free living or an activity protocol) in people with a chronic disease [22-24]; however, these studies only tested noncommercially available activity trackers and mainly evaluated energy expenditure instead of step count. Results from other studies with participants with chronic diseases are not generalizable to daily practice because they did not have step count as the primary outcome (eg, mostly energy expenditure) [25-31], involved only walking and no other activities of daily living [32-35], or free living conditions were not protocoled (eg, cardiac patients [36] and patients with COPD [37] or cancer [38]).

As stated before, for both people with a chronic disease and their therapists, insight into physical activity level and patterns outside of therapy are very relevant. Since no article was found that matched our criteria, we decided to validate 9 potential trackers ourselves in people with a chronic disease.

The main aim of this study was to investigate the validity of 9 selected commercially available activity trackers for measuring step count in people with a chronic disease receiving physiotherapy during a selected set of activities of daily living. Results from this study should provide guidance in choosing the right activity tracker for people with a chronic disease.

## Methods

### Study Design

A cross-sectional validity study with 9 activity trackers was performed in patients with chronic diseases. The data collection took place over a 1-year period. All participants provided written informed consent. This study was approved by the local ethics boards (Atrium-Orbis-Zuyd Medical Ethical Committee, 15-N-48; Adelante Medical Ethical Committee, MEC-15-07).

### Participants

Participants were recruited from 2 physiotherapy practices (Fysiotherapie Schaesberg and ParaMedisch Centrum Zuid) and a rehabilitation center (Adelante Zorggroep) in the Netherlands. Patients were included if they were aged 18 years and older and diagnosed with at least 1 of the following chronic diseases: cardiovascular disease, COPD, diabetes mellitus, chronic pain, cancer, or osteoarthritis. Exclusion criteria were insufficient understanding of the Dutch language, use of a walking aid, and asymmetrical gait (eg, stroke). A power calculation was conducted, and a minimum of 57 participants with an equal spread among the 6 chronic subpopulations was considered to be sufficient for a validity study [39].

### Activity Trackers

Researchers and physiotherapists agreed to the following selection criteria for commercially available activity trackers: costs less than €150 (US $185), no monthly costs for a subscription, real-time feedback on the tracker to the user, measures number of steps, and no chest strap to perform heart rate measurements. To ensure that the scope of different system requirements was covered, trackers were randomly selected in a second round based on the following criteria: a variety of wearing places (eg, belt, wrist) and types of activity trackers (eg, pedometers, accelerometers). Hence, 9 activity trackers were selected: Accupedo (Corusen LLC), Activ8 (Remedy Distribution Ltd), Digi-Walker CW-700 (Yamax), Fitbit Flex (Fitbit Inc), Lumoback (Lumo Bodytech), Moves (ProtoGeo Oy), Fitbit One (Fitbit Inc), UP24 (Jawbone), and the Walking Style X (Omron Healthcare Europe BV) (Table 1).

### Data Collection and Procedure

Participants were measured in either of the physiotherapy practices or the rehabilitation center. Baseline characteristics were reported (gender, age, body weight, height, diagnosed chronic disease) by 1 of the 10 participating physiotherapists or a psychologist. For participants with COPD, the Global Initiative for Chronic Obstructive Lung Disease stage [40] was specified. For participants with osteoarthritis, a differentiation was given for lower extremity (toe, ankle, knee, hip), upper extremity (finger, wrist, elbow, shoulder), and cervical and lower spine. In participants with cancer, curative and palliative treatments were distinguished. Two questionnaires were completed with the participant. The Cumulative Illness Rating Scale (CIRS) was used to indicate the number and severity of comorbidities [41,42]. For an impression of the participant’s physical activity level, a brief physical activity assessment tool was used to determine whether the participant was sufficiently active [43]. After completing the questionnaires a 10-meter walk test (10MWT) was performed 3 times to determine the average comfortable walk speed of the participant [44]. Thereafter, participants were fitted with 3 or 4 activity trackers, chosen at random, and asked to perform the activity protocol.

### Activity Protocol

Tasks representing activities of daily living from protocols in previous validation studies [24,29,45,46] were used to create the protocol for this study (Table 2). In order to match the participants’ physical activity capacity, 2 versions of the protocol were developed, assuming that the length of the protocol had no influence on the validity of the trackers. The short version of the protocol did not include lying on a bed, vacuum cleaning on the spot, and 3 additional periods of standing, shortening the execution time of the protocol by 9 minutes. Activity trackers not able to classify different postures were used in the short protocol. Participants were given extra resting periods during the protocol of they needed them.

Step count was collected from the activity trackers before and directly after the protocol. The entire activity protocol was recorded on video camera, focusing only on the lower extremity for privacy reasons. The video recordings were used to determine the number of steps taken by each participant. Step count was manually counted using a digital step counter (gold standard). A person was considered to make a step when the entire foot was lifted from the floor and was placed back on the floor again (detailed information is published elsewhere [39]). The 7 raters involved used a standardized written assessment protocol and were trained by 1 researcher beforehand. The first 2 video recording assessments per rater were checked by the researcher (DU) to secure standardization of the measurement method.

### Data Analysis

Data analysis was performed using the SPSS Statistics version 23.0 (IBM Corp). Descriptive statistics of the participant characteristics were presented as raw data and percentages for the categorical variables gender, diagnosed disease, and physical activity (sufficient/insufficient) [43] and as means and standard deviations for the continuous variables age, CIRS score, and average walk speed.

The video recordings of the activity protocols were analyzed by at least 1 researcher. One-tenth randomly chosen video recordings were analyzed by a second researcher to assess intra observer reliability of our gold standard. This was assessed by intraclass correlation coefficients (ICCs; 2-way random, absolute agreement) and Bland-Altman plots including limits of agreement [47]. It was hypothesized that there would be a strong correlation (*r*>.90) [48].

**Table 1:** Selected commercially available activity trackers used in this validity study.

Activity tracker	Manufacturer	Type	Wearing position	Outcome variables
Accupedo	Corusen LLC	App	Belt	Number of steps
Activ8	Remedy Distribution Ltd	Accelerometer	Trouser pocket	Number of steps; time spent lying, sitting, standing, walking, running, and cycling; active minutes
Digi-Walker CW-700	Yamax Corp	Pedometer	Wrist	Number of steps, active minutes
Flex	Fitbit Inc	Accelerometer	Wrist	Number of steps, active minutes
Lumoback	Lumo Bodytech	Accelerometer	Lower back	Number of steps; time spent lying, sitting, standing, walking, running, and cycling; active minutes; number of sit-to-stand transitions
Moves	ProtoGeo Oy	App	Trouser pocket	Number of steps, active minutes
One	Fitbit Inc	Accelerometer	Belt	Number of steps, active minutes
UP24	Jawbone	Accelerometer	Wrist	Number of steps, active minutes
Walking Style X	Omron Healthcare Europe BV	Pedometer	Belt	Number of steps, active minutes

**Table 2:** The developed activity protocol based on principles and free living tasks from other protocols.

Activity type	Duration of activity, repetitions, or walking distance	Included in short version
Standing	1 minute	Yes
Simulated cleaning of windows	1 minute	Yes
Walking weaving around cones	7 meters	Yes
Sitting in a chair	2 minutes	Yes
Standing	1 minute	No
Vacuum cleaning on the spot	1 minute	No
Vacuum cleaning while walking	1 minute	Yes
Walking weaving around cones	7 meters	Yes
Walking up and down stairs (3 or 4 steps)	3 times	Yes
Lifting a 1-kg object and placing it on a table	1 minute	Yes
Walking in a straight line	7 meters	Yes
Lying in a bed	6 minutes	No
Sitting in a chair	5 minutes	Yes
Standing	1 minute	No
Walking in a straight line while carrying a shopping bag (2.5 kg)	7 meters 2 times	Yes
Walking sideways along a 2-meter kitchen counter	2 ways 3 times	Yes
Standing	30 seconds	No
Walking in a straight line	7 meters	Yes
Cycling (50 to 60 rpms^a^ at 30 watts)	3 minutes	Yes
Total time	28 to 33 minutes	19 to 24 minutes

^a^Revolutions per minute.

The validity of the activity trackers was assessed in multiple ways. To gain insight into step count distribution, descriptive statistics and scatterplots were used for all trackers. To gain insight into the strength of the relation between measured steps by the activity trackers and the gold standard, Pearson correlation coefficients were calculated. It was hypothesized that there would be at least a moderate correlation (*r*>.40) [48]. To assess systematic differences between the activity trackers and the gold standard, paired samples *t* tests were used. With a power of 80%, a *P* value below .05 was considered to be of statistical significance. To examine the level of agreement between the activity trackers and the gold standard, Bland-Altman plots were constructed with their associated 95% limits of agreement [49].

To assess if there were difference between the chronic diseases, visual inspection of the scatterplots were performed. To assess if there were systematic differences between the average mean differences of the short and long protocols, independent *t* tests were used. To test if there was a systematic difference in the mean difference between the gold standard and the activity tracker between the short and long protocols, a paired sample *t* test was used in the case of normally distributed data. In the case of missing data, pairwise deleting was applied.

## Results

### Participant Characteristics

A total of 130 participants with chronic diseases participated in this validation study (Table 3). Cardiovascular disease, chronic pain, and osteoarthritis were the most prevalent single conditions, and 26.4% (34/130) of the population had multimorbidity. The combinations occurring most often were osteoarthritis with chronic pain (6/34, 17.6%), osteoarthritis and diabetes (4/34, 11.7%), and COPD and diabetes (3/34, 8.8%). Approximately 60% (75/130) of the participants were sufficiently physically active in their daily life according to the physical activity assessment tool. Of the included COPD patients, 7.7% (1/14) were diagnosed with stage 1 COPD, 35.7% (5/14) with stage 2, 42.9% (6/14) with stage 3, and 14.3% (2/14) with stage 4. Of the cancer patients, 82.6% (19/23) had a curative treatment and 17.4% (4/23) a palliative treatment. The affected joints in osteoarthritis were almost equally spread in upper extremity (22/33, 66.7%), spine (24/33, 72.7%), and lower extremity (23/33, 69.7%). There were 2 missing values for gender, diagnosed disease ,resting heart rate and body mass index (BMI) (2/130, 2.6%), and 3 missing values for age (3/130, 3.9%). There was 1 missing value for the number of steps from the Lumoback (1/51, 5.1%) and 1 from the Accupedo (1/50 5%).

**Table 3:** Characteristics of the included population.

Characteristics		Participants (n=130)
Gender, male, n (%)		55 (43.6)
Age, years, mean (SD)		61.5 (11.1)
Body mass index, kg/m^2^, mean (SD)		27.7 (5.2)
**Blood pressure, mm Hg, mean (SD)**		
	Systolic	136.2 (20.3)
	Diastolic	80.3 (9.7)
Resting heart beat, beats per minute, mean (SD)		74.0 (12.2)
Transcutaneous oxygen saturation, %, mean (SD)		96.4 (2.3)
**Diagnosed disease, n (%)**		
	Cardiovascular disease	20 (15.2)
	Chronic obstructive pulmonary disease	15 (11.4)
	Diabetes mellitus	8 (6.1)
	Cancer	15 (11.4)
	Osteoarthritis	18 (14.4)
	Chronic pain	19 (14.4)
	Combination	34 (27.3)
Comorbidity, CIRS^a^ 0 to 52, mean (SD)		6.2 (3.9)
Average walk speed^b^ (m/s) mean (SD)		1.3 (0.3)
**Sufficient total activity, n (%)^c^**		74 (56.4)
	Physical activity level (0 to 8), mean (SD)	3.8 (2.4)
	Physical activity with moderate intensity (0 to 4), mean (SD)	1.6 (1.6)
	Physical activity with vigorous intensity (0 to 4), mean (SD)	2.2 (1.5)

^a^CIRS: Cumulative Illness Rating Score.

^b^Based on the 10-meter walk test [44].

^c^Based on the brief physical activity assessment tool and its accompanying cut-off value [43].

### Interobserver Reliability

The interobserver reliability of the gold standard, calculated in the random sample, was high (ICC_agreement_ 0.98, *P*<.001, 95% CI 0.96 to 0.99). There was no substantial offset (SEM_agreement_ = 81.6) and the Bland-Altman plots showed no systematic differences between the observers (with narrow limits of agreement: –35.3 to 30.8 steps).

### Step Count

**Figure 1:**
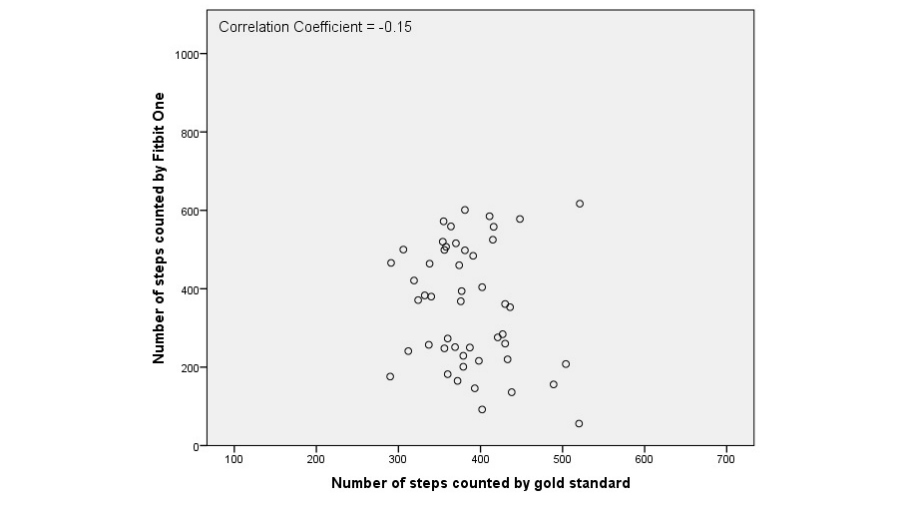
Scatterplot of the number of steps counted by Fitbit One and the gold standard.

**Figure 2:**
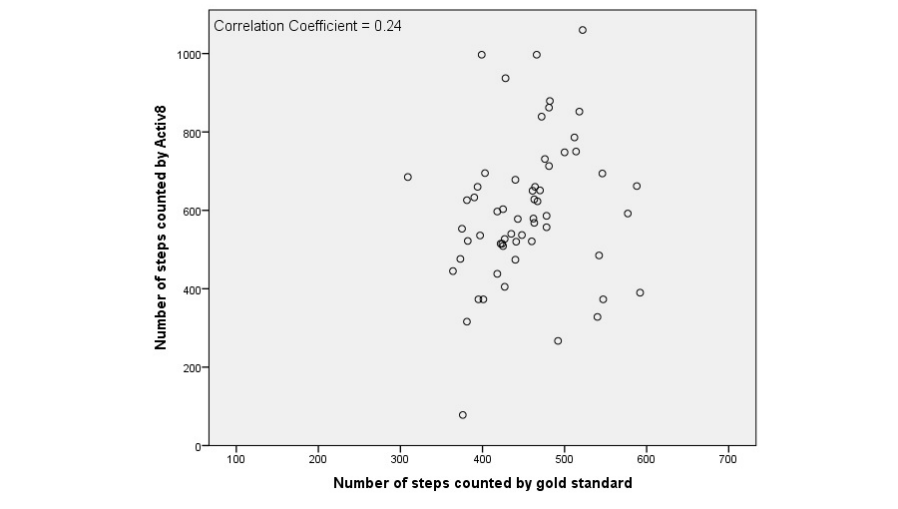
Scatterplot of the number of steps counted by Activ8 and the gold standard.

**Figure 3:**
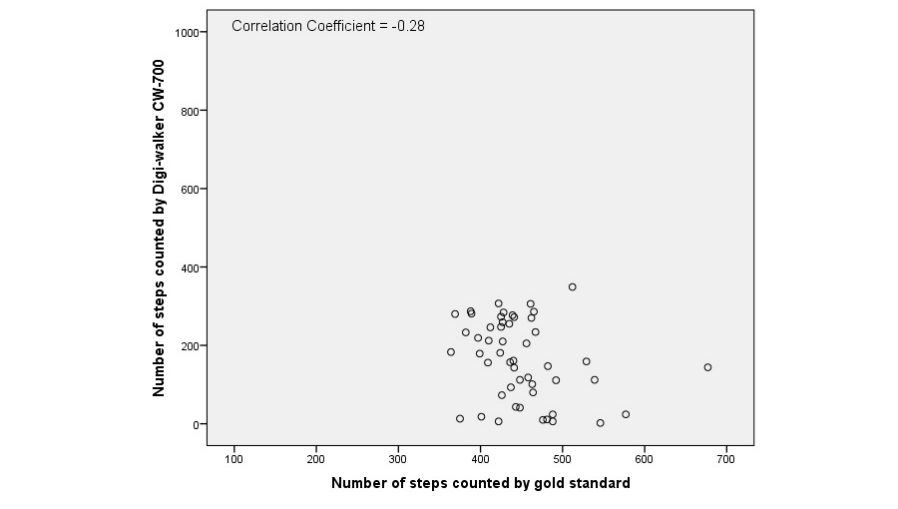
Scatterplot of the number of steps counted by Digi-walker CW-700 and the gold standard.

Step count for the gold standard and each tracker are shown in Table 4. The average total number of steps during the short and long activity protocols counted by the gold standard was 405.4 (SD 84.7). The average total number of steps for the short protocol was 327.7 (SD 54.3) and the average total number of steps for the long protocol was 446.6 (SD 58.6). There was no significant difference between the mean difference (gold standard versus activity tracker) in the short and long protocols. For all activity trackers except for the Activ8, the mean difference with the gold standard was lower than zero, which indicated an underestimation of the total number of steps. The mean difference between the tracker and gold standard varied from –29.7 (SD 155.10) for the Fitbit One to 252.4 (SD 129.0) for the Digi-Walker CW-700. Overall, data distribution showed a wide range of observations for all activity trackers. There were no differences found per chronic disease compared to the whole population. Scatter plots of the Fitbit One, Digi-Walker CW-700, and Activ8 are presented in Figures 1-3 to give examples of data distribution.

### Strength of the Relation and Systematic Difference

The correlation between the number of steps measured by the activity trackers and the gold standard was weak for all activity trackers ranging from *r*=–.02 for the Moves to *r*=–.33 for the Digi-Walker CW-700 (Table 5). The average underestimation of all trackers and the average overestimation of the Activ8 revealed a significant systematic difference with the gold standard for step count, expect for the Fitbit One (*P*=.35).

**Table 4:** Descriptive statistics of step count by activity tracker compared to the gold standard.

Activity tracker	Number of participants	Average mean difference in step count (SD)^a^	Average median difference in step count (25 to 75 percentile)^a^	Limits of agreement (lower bound–upper bound)
Accupedo	50	–176.3 (132.1)	–174.5 (–251.0 to –102.5)	–435.2 to 82.7
Activ8	62	107.3 (251.9)	126.0 (30.5 to 243.5)	–471.3 to 721.0
Digi-Walker CW-700	52	–284.5 (129.0)	–253.0 (–383.0 to –169.0)	–537.4 to –31.7
Fitbit Flex	47	–93.5 (126.7)	–111.0 (–167.0 to 3.0)	–326.9 to 123.7
Lumoback	51	–178.5 (96.0)	–168.0 (–205.5 to –117,0)	–366.9 to 9.3
Moves	48	–146.6 (216.3)	–215.0 (–279.5 to –89.3)	–570.5 to 277.4
Fitbit One	49	–29.7 (155.1)	–8.0 (–160.0 to 128.0)	–367.8 to 308.6
UP24	49	–252.4 (104.7)	–266.0 (–327.0 to –176.5)	–457.7 to –47.2
Walking Style X	50	–204.4 (117.7)	–206.5 (–256.0 to –105.0)	–438.0 to 27.2

^a^Activity tracker minus gold standard.

### Level of Agreement

**Figure 4:**
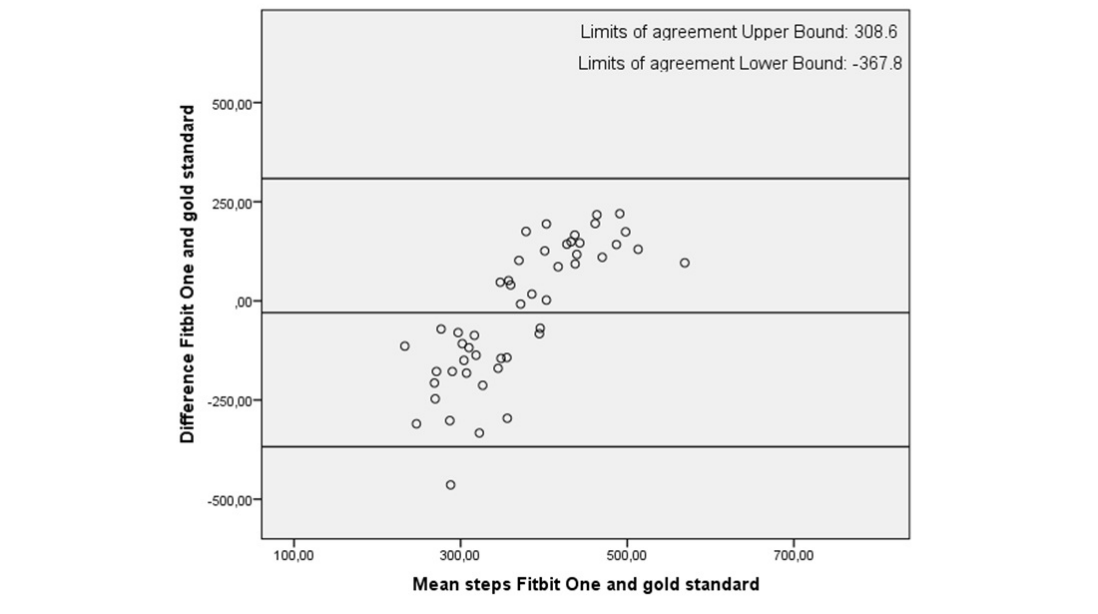
Bland-Altman plot of Fitbit One and the gold standard.

**Figure 5:**
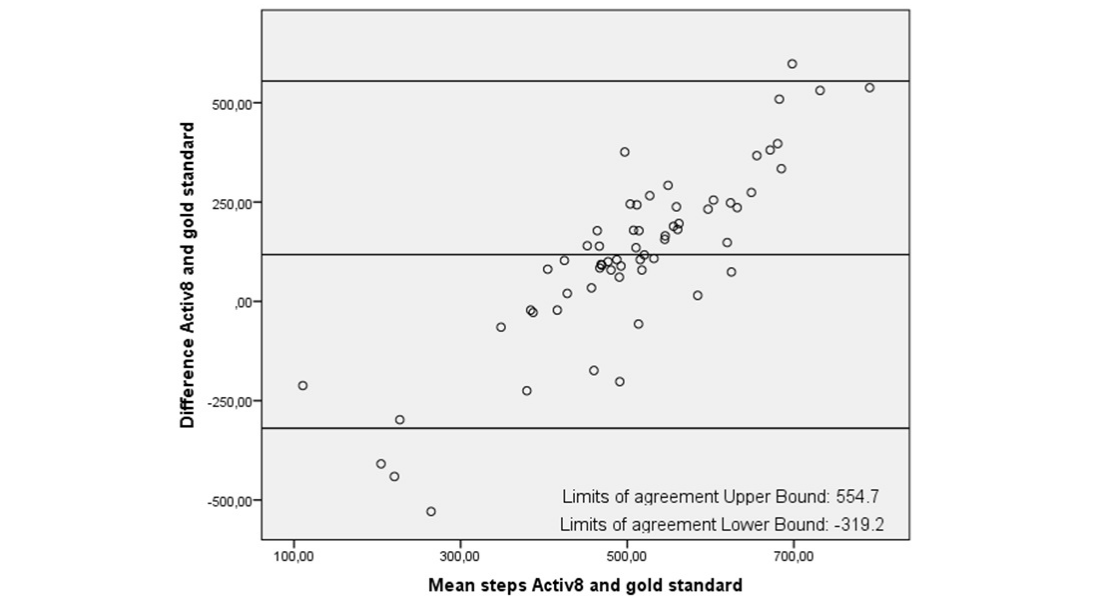
Bland-Altman plot of the Aciv8 and the gold standard.

**Figure 6:**
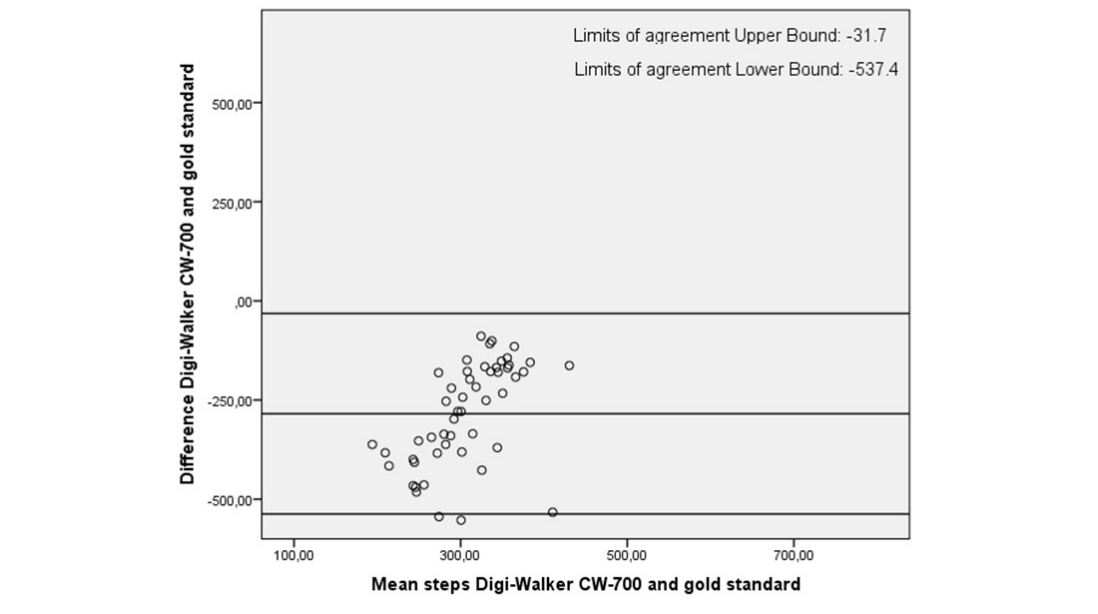
Bland-Altman plot of Digi-Walker CW-700 and the gold standard.

In all plots the limits of agreement are high, with the highest limits of agreement (–471.3 to 721.0) for the Activ8 (Table 4). In the plots, 2 trends are visible: either an over- and underestimation of the number of steps during the activity protocols as shown in Figures 4 and 5 (eg, Fitbit One and Activ8) or an underestimation of the number of steps only, as shown in Figure 6 (eg, Digi-Walker CW-700). Depending on the height of step count, overestimation or underestimation was shown. Overestimation became more pronounced when participant took more steps and vice versa.

### Systematic Difference Between Short and Long Protocols

Only the Walking Style X, Accupedo, and Fitbit Flex were used in both protocols. For all trackers, there were no systematic differences found for the average mean difference in step count between the short and long protocols.

**Table 5:** Correlation coefficient of the activity trackers and the gold standard for step count.

Activity tracker	Correlation coefficient (*P* value)	t value (*P* value)
Accupedo	.32 (.02)	–9.4 (<.001)
Activ8	.24 (.06)	–3.9 (.001)
Digi-Walker CW-700	–.33 (.02)	–6.2 (<.001)
Flex	.31 (.04)	–5.1 (<.001)
Lumoback	.19 (.20)	–6.2 (<.001)
Moves	–.02 (.88)	–3.4 (.001)
One	–.15 (.30)	–0.9 (.35)
UP24	.09 (.52)	–6.9 (<.001)
Walking Style X	.25 (.08)	–12.3 (<.001)

## Discussion

### Principal Findings

The results of this study showed that none of 9 selected commercially available activity trackers was valid for measuring steps while individuals engage in activities of daily living among a diverse group of patients with various chronic diseases receiving physiotherapy in the Netherlands.

All activity trackers in this study had an average underestimation in step count except the Activ8, which overestimated step count. The Digi-Walker CW-700 and Lumoback consistently underestimated step count in every participant, while the other activity trackers had a combination of under- and overestimation. For all trackers, the correlations between step count measured by the activity trackers and the observed steps were low. On group level, the Fitbit One seemed to be the best activity tracker due to its low mean difference; however, on individual basis the scatter and Bland-Altman plots showed a large under- and overestimation in step count.

Several studies have shown that a low walking speed decreases the validity of activity trackers [12,16,50,51]. For an activity tracker to measure the number of steps correct, a walking speed of 0.8 m/s is required. All of our participants walked faster than 0.8 m/s during the 10MWT; therefore, their walking speed should have been sufficient for an accurate measurement by the activity tracker. However, the activity protocol consisted of different household tasks such as vacuum cleaning and washing windows, resulting in a walking speed below 0.8 m/s. Recently, Nelson et al [52] published the results of a validation study in which the Fitbit Flex and Fitbit One were assessed during activities of daily living in a healthy population. They concluded that these activity trackers underestimate step count by 60% during household activities, but during walking activities the percentage error was within 4%. Nelson et al concluded that this difference might come from slow ambulation speed and shuffling during these household activities. Although the populations differ, these results confirm the findings in our study. Our standardized activity protocol was based on earlier protocols with activities of daily living in COPD patients [24,29,45] and is therefore comparable to real-life performance of people with a chronic disease. Our protocol consisted of various activities of short duration, since this is more comparable to the performance of the activities in the daily life of people with a chronic disease. Since the study population had a limited physical activity capacity and more fatigue, pain, and possibly dyspnea, the requirements of the longer protocol might not have matched their physical possibilities and might not represent the daily life of people with a chronic disease. During the execution of the study, all patients were able to perform the entire protocol, and no patients had to be excluded due to the effort required by the protocol. However, the results of our study contradict studies performed in healthy populations in which the 9 tested activity trackers showed good validity in free-living situations [19,20]. An explanation could be that the walking speed is faster during free-living situations because patients perform more walking activities in comparison to an activity protocol with activities of daily living. To the authors knowledge, only 1 validation study was performed in people with a chronic disease (cardiac patients) using one of the assessed activity trackers (Fitbit Flex [36]). This study concluded that there was a high correlation between the Fitbit Flex and the Actigraph for step count (*r*=.95).

### Limitations and Strengths

The chosen activity trackers were the most up-to-date activity trackers at the time. During this study, several updates were released for the chosen activity trackers (mostly the exterior instead of the algorithm), and several new activity trackers were brought to the market. But the chosen activity trackers are still the most popular and most used activity trackers currently available [53-55].

In this study design, 2 activity protocols were used. It was assumed that the length of the protocol had no influence on the trackers’ validity because the removed activities were activities that didn’t require walking. There were no systematic differences in average mean difference in step count between the short and long protocols.

For determining the validity of the step count, the definition of a step is very important. In this study, a step was defined as when the entire foot was lifted from the floor and placed back on the floor again. However, shuffling is frequently seen in elderly populations and in people with a chronic disease [12]. If shuffling steps were included in our analysis (thus more steps during the protocol), more underestimation of the activity trackers would be likely, implying an even lower validity.

In this study, it wasn’t possible to report validity of the activity trackers per activity. All selected activity trackers were commercially available trackers, and thus their algorithms and time slots were not available on request. Without specific information regarding (at least) the timeslots, it was not possible to disentangle time per activity.

In this study, we used different methods for evaluation of the validity. By using these different methods, insight was gained on validity on both group and individual levels. Validity on individual level is important for daily practice for patients and therapists. We included the *P* value for the correlation coefficient; however, this is a measurement on group level and not on individual level. Therefore, the significant correlations are not clinically relevant. Moreover, the 3 significant correlations (Accupedo, Digi-Walker CW-700, and the Flex) are still considered weak correlations [48].

A strength of this study is the use of observed steps as gold standard. The high reliability of this gold standard assures very little systematic bias in the analysis method. The chronic diseases included in this study are those most frequently seen by physiotherapists in the Netherlands [2], implying that the study results might be generalizable to a broad population. However, this should be confirmed by including a broader range of patients with chronic diseases not limited to primary care physical therapy practices.

### Clinical Relevance

Guidelines recommend objectively measuring the physical activity level of a patient outside of guided therapy [2]. However, underestimation or overestimation of physical activity by an activity tracker is not desirable. Not only might it demotivate people to engage in physical activity, it may also influence the advice and intervention of physiotherapists. This study showed that the trackers are not valid for activities of daily living performed in this study. Considering this limitation, the trackers should only be used to measure steps during free living situations in which patients perform more walking activities.

### Conclusion

This study showed that the validity of 9 commercially available activity trackers is low measuring steps while individuals engage in activities of daily living among a diverse group of patients with various chronic diseases receiving physiotherapy. Frequent underestimation and a wide range of measurements were seen for step count during a protocol with activities of daily living compared to observed steps as gold standard.

## Abbreviations

10MWT: 10-meter walk test
CIRS: Cumulative Illness Rating Scale
COPD: chronic obstructive pulmonary disease
ICC: intraclass correlation coefficient
